# Surface Modification of Biomaterials: A Quest for Blood Compatibility

**DOI:** 10.1155/2012/707863

**Published:** 2012-05-27

**Authors:** Achala de Mel, Brian G. Cousins, Alexander M. Seifalian

**Affiliations:** ^1^UCL Centre for Nanotechnology & Regenerative Medicine, University College London, Pond Street, London NW3 2QG, UK; ^2^Royal Free Hampstead NHS Trust Hospital, Pond Street, London NW3 2QG, UK

## Abstract

Cardiovascular implants must resist thrombosis and intimal hyperplasia to maintain patency. These implants when in contact with blood face a challenge to oppose the natural coagulation process that becomes activated. Surface protein adsorption and their relevant 3D confirmation greatly determine the degree of blood compatibility. A great deal of research efforts are attributed towards realising such a surface, which comprise of a range of methods on surface modification. Surface modification methods can be broadly categorized as physicochemical modifications and biological modifications. These modifications aim to modulate platelet responses directly through modulation of thrombogenic proteins or by inducing antithrombogenic biomolecules that can be biofunctionalised onto surfaces or through inducing an active endothelium. Nanotechnology is recognising a great role in such surface modification of cardiovascular implants through biofunctionalisation of polymers and peptides in nanocomposites and through nanofabrication of polymers which will pave the way for finding a closer blood match through haemostasis when developing cardiovascular implants with a greater degree of patency.

## 1. Introduction

**Figure 1:**
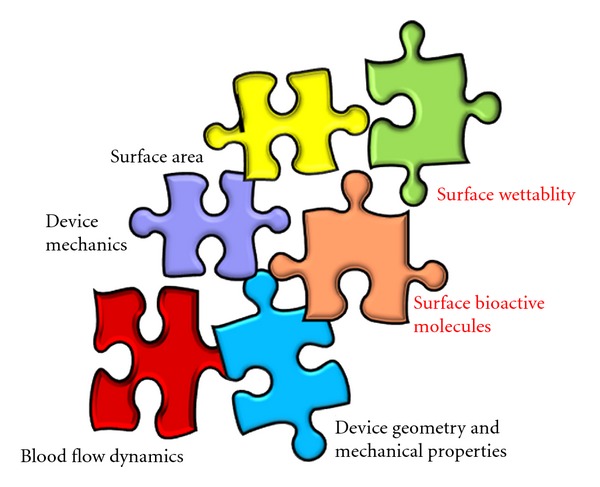
Haemocompatibility-determining factors in a cardiovascular device; marked in red are areas of interest in this paper.

Cardiovascular disease accounts for a significant percentage of mortality and morbidity in the ageing population and has an estimated increase in the coming years [1]. There is an urgent clinical need for improved cardiovascular devices, which mainly include vascular bypass grafts, vascular stents, and heart valves, which will promote desirable blood-biomaterial interactions with a high patency. Vascular occlusive disease holds the greatest risk factor most emphasised in the coronary arteries where cardiac ischemia may lead to complete heart failure. Main reperfusion-based surgical intervention options for these diseases involve angioplasty, stenting, endarterectomy, and bypass graft surgery depending on the degree of occlusion. Cases with greater than 70% occluded arteries are required to be treated with bypass grafts. For small diameter bypass grafts, autologous bypass conduits are preferred for primary revascularisation [2]. However, 3–30% patients are presented with no autologous vessels due to previous disease conditions and thus there is a need for vascular grafts which could perform closely to autologous vessels [3]. Graft thrombogenicity due to material surface incompatibility and altered flow dynamics at the site of anastomosis or distal outflow are recognised as primary reasons for blood contacting device failure [4]. There is a great interest in research strategies that focus upon surface techniques by modifying the physicochemical properties at the implant surface [5] and by combining a biomimetic approach through functionalisation which presents an exciting challenge to improve patency rates clinically (Figure 1). This paper aims to review some of the significant approaches in modifying a material surface to create optimal interactions with blood.

## 2. Blood-Implant Surface Interactions: Thrombogenicity

**Figure 2:**
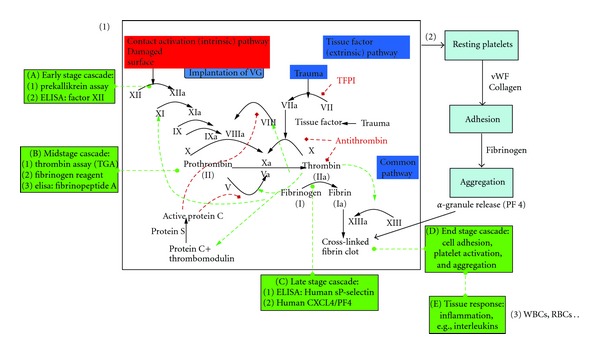
Intrinsic pathway of blood coagulation: highlighted are the main factors which are involved in blood coagulation. Numbered events distinguish as (1) biochemical, (2) platelets, and (3) whole blood (red and white blood cells). Image is adapted from http://en.wikipedia.org/wiki/File:Coagulation_full.svg.

The initial events leading to thrombosis surrounding the tissue-implant interface are mediated by surface interactions with adsorbed proteins (intrinsic pathway) or through the release of tissue factor (TF) from damaged cells at the site of injury (extrinsic pathway) [6] (Figure 2). The intrinsic pathway is independent of injury. Adsorbed surface proteins form a complex composed of collagen, high molecular weight kininogen (HMWK), prekallikrein, and factor XII. Inactive precursors (clotting factors) change conformation and are converted into active enzymes via a biochemical cascade resulting in platelet activation (with the aid of additional cofactors). Cleavage of prothrombin via the prothrombinase complex bound to cellular membranes generates thrombin, and by converting fibrinogen to fibrin, forms a stable insoluble gel (red thrombus or clot).

Vascular injury and damage to the endothelium releases TF, collagen, and von Willebrand factor (vWF) to initiate the extrinsic pathway. Clotting factors interact with platelet surface receptors and play a fundamental role in the interaction of collagen to initiate thrombosis, release growth factors and cytokines to enhance the coagulation cascade and strengthen the haemostatic plug. The platelets change morphology and agglomerate forming a thrombus layer. It is important to note that both pathways converge during the formation of the prothrombinase complex leading to thrombin generation referred to as the common pathway.

Vascular procedures such as arteriovenous graft placement and angioplasty damage the adventitial and medial tissues of the arterial wall with injury to the endothelium lining the intima [4]. For example, angioplasty is a controlled traumatic event, which is aimed at causing plaque rupture by widening a narrowed or obstructed vessel. These processes can expose otherwise intact subendothelial matrix removing the protective endothelium and expose medial smooth muscle cells (SMC) directly to blood flow, and other procoagulants and proinflammatory blood constituents. Tissue trauma rapidly initiates the recruitment of inflammatory cells that release potent cytokines and promote SMC migration and proliferation. The anticoagulant and vascular protective functions of intact endothelium from prostacyclin (PGI_2_) and nitric oxide (NO) required for the regulation of blood flow soon diminish [7]. Both molecules are necessary to inhibit platelet adhesion, aggregation and activation to the endothelium and SMC, which are considered early events in the development of intimal hyperplasia (IH). Furthermore, NO inhibits SMC proliferation and migration. In addition, the adventitial layer is partially removed for creating the anastomosis during surgery depriving the vessel wall of oxygen and vital nutrients [8].

Almost all materials are considered to be thrombogenic with the exception of the endothelial cell (EC) layer, which lines the vasculature. Large diameter vascular grafts were originally thought to be antithrombogenic in nature. For example, expanded polytetrafluoroethylene (ePTFE) bypass grafts appear nonthrombogenic due to the high flow rates of blood past the luminal surface, but in reality, all are thrombogenic to a certain degree.

In healthy individuals the flow of blood is laminar but when compared with diseased or occluded arteries may often be transitional or even turbulent in behaviour. At the blood-biomaterial interface, haemodynamic forces of shear stress at the wall surface play a critical role in blood contacting devices and influence protein adsorption [9], platelet and leukocyte adhesion. Leukocytes recognise specific proteins and adhere under flowing conditions to initiate further cell signalling and recruitment events. A study evaluating leukocyte adhesion on polyurethanes materials has shown that cell density decreased with increasing shear stress. Certain shear stress models have been studied (particular when applied to seeded vascular grafts) to promote EC retention and found to correlate with changes in the EC phenotype [10]. Various strategies exist to inhibit these processes and prolong graft patency, including modification of grafts with various anticoagulants (heparin), antiplatelet factors (glycoprotein IIb/IIIa inhibitors), and antiproliferating agents (rapamycin). In the following sections we consider different surface modification techniques that are designed to minimise complications that arise at the blood-biomaterial interface.

## 3. Role of Proteins in Optimal Blood-Biomaterial Interactions

Cardiovascular implants, in the body, are subjected to the “Vroman effect” [11] which highlights the dynamic interactions with water and proteins to synthetic material. This event is rapid (<1 sec), leading to the formation of a thin protein film in the order of nanometers in thickness [12]. The adsorption of proteins (composed of polar, nonpolar, and charged side groups) contributes to the surface activity. Once present at the surface, protein molecules interact with water, electrolytes, and the underlying surface chemistry (and energy) of the material through hydrogen bonding, van der Waals, pi-pi (*π*-*π*) stacking, and electrostatic interactions. Exactly which force governs the interaction of proteins on surfaces depends upon the particular protein and other factors including size, charge, conformation, and unfolding rate described by Vroman [13]. Chemical and physical properties of the materials, for example, surface chemistry, energy (charge) and topography, influence the interfacial behaviour adjacent to the biomaterial. The interfacial region at the blood-biomaterial surface continually alters and redistributes the protein/electrolyte/water layer, and the host cells and tissues react to changes in this layer. Material surfaces with zero interfacial energy and reduced enthalpic and entropic effects do not strongly support cell/thrombin adhesion [14]. Surface wettability of a biomaterial is highly significant and, in addition to differential protein adsorption, platelets respond differently to hydrophobic or hydrophilic monomers [15].

Adsorption of plasma and extracellular matrix (ECM) proteins (fibrinogen, albumin, and *γ*-globulin), and to a lesser degree fibronectin, collagen, vWF, coagulation factors XI and XII, and HMWK play a crucial role in balancing thrombosis and haemostasis [16]. Such proteins direct and aid the adhesion of red blood cells, platelets (the first cellular components to adsorb to the protein film), followed by leukocytes, and EC. The cellular components interact with the protein layer to guide migration, initiate blood coagulation, and stimulate cell proliferation and differentiation, as specific proteins present binding sites for macromolecules and receptors guiding the recruitment of further cells interacting within the vasculature.

**Figure 3:**
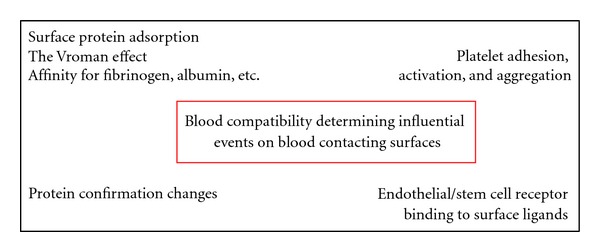
Main mechanisms influencing blood compatibility.

Protein adsorption and subsequent cell attachment and behaviour in response to an implanted foreign material is determined by a variety of material properties including surface chemistry, topography, dissolution rate, and the micro-/macromechanical elasticity. Material surface properties can therefore be modified by physicochemical modification and/or biofunctionalisation to promote desirable protein and cellular interactions.  Figure 3 summarizes the main mechanisms, which influence blood compatibility.

## 4. Surface Modification of Blood Contacting Materials

Much effort has focused on surface modification to optimise antithrombogenic surface properties and two approaches exist in the development of cardiovascular grafts. The first approach involves the design of a permanent vascular replacement, which has a nonadhesive, inert, nonbiofouling surface. Physicochemical methods have been applied to achieve this aim using electrochemical polishing, surface roughening, ordered patterning, plasma treatment [17], chemical etching, and passive or covalent surface coatings. The second approach aims to functionalise the grafts in such a way that it facilitates (or activates) a cascade of biological events which eventually regenerates or replaces functioning tissue. Biofunctionalisation of surfaces is a popular research theme, which relies on the tools of biology to create biomimetic surfaces to incorporate biologically active (or inactive) molecules to generate specific response(s) [18–22].

**Figure 4:**
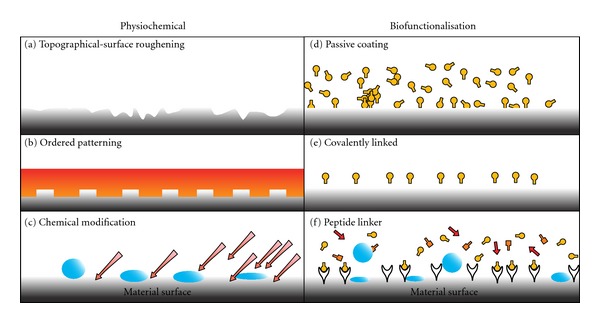
Examples of various physical, chemical, and biofunctionalisation techniques to enhance haemocompatibility. Biofunctionalised surfaces interact with cell surface receptors, that is integrins. Whereas physiochemical modification can influence cell-material interactions through charge, topography, and attractive/repulsive forces due to hydrophobic and hydrophilic interactions [26].

Figure 4, Table 1 present a summary of the principle methods in applied surface modification techniques. In this way, surface modification can be directed towards optimising the following: (1) protein adsorption (2), the generation of thrombin (and its formation leading to blood coagulation), (3) platelet adhesion (followed by aggregation and activation), and (4) cellular behaviour at the surface of the prosthesis. All strategies are designed to optimise patency-limiting thrombogenic events at the blood-biomaterial interface. For example, vascular graft endothelialisation has been highlighted as the ultimate solution to address thrombogenicity, and its associated complications.

### 4.1. Physicochemical Modification

A range of physical techniques has been applied to modify the surface topography of vascular graft materials. Topography on the micron and nanometre scale is an important physical property, which influences protein adsorption, platelet adhesion, thrombogenicity, and cell behaviour [23]. The inclusion of pores, pits, and groves become unavoidable at this scale during the manufacturing process of blood contacting devices. For example, a recent study revealed that the surface roughness of ePTFE graft luminal surfaces was significantly higher (147.0 nm) when compared with external surfaces (1.74 nm). Plasma proteins such as fibrinogen have been shown to adhere to nanostructures and bind to platelet receptors more efficiently than flat structures [24]. Albumin, fibrinogen, and fibronectin all interact with a dialysis membrane's surface topography, which plays a crucial role in the adsorption process. Such surfaces have been shown to promote fibronectin and vitronectin adsorption and direct a cascade of interactions from the blood and surrounding tissues. Surface porosity is a crucial factor when considering the topography of vascular graft materials [25]. A recent study looked at the effect of porosity (ranging from 5 to 90 *μ*m in diameter) on EC growth. It was found that EC cell growth was enhanced by smaller pores (5–20 *μ*m in diameter) and at a lower interpore distances.

Changing the surface topography on the micron and nanometre scale also lead to localised changes in surface chemistry as both physicochemical cues are intrinsically linked. The primary aim of topographical and chemical surface modification is to encourage desirable protein, cellular, and tissue interactions at the blood-biomaterial interface, thus improving patency and performance of the material, since all are known contributory factors that influence thrombogenicity. Nanocomposite materials are recognisedto offer favourable solutions as biomaterials for cardiovascular implants. Nanocomposite polymers in general have found to be amphiphilic, thermodynamically stable and, when used in vascular bypass graft development, they have shown to exert novel advantageous properties such as favourable blood response [27], biostability [28], and enhanced mechanical properties compared to grafts with conventional material. While being viscoelastic, polyhedral-oligomeric-silsesquioxane-poly(carbonate-urea)urethane (POSS-PCU) has been shown to have strength similar to natural arteries. POSS-nanocomposite polymer, used for cardiovascular implants, which include vascular bypass grafts, stents, and heart valves has been proved to have antithrombogenic properties [29].

Nonfouling surfaces have been used to prevent protein adsorption and platelet adhesion. Much effort has focused upon the passivation of materials using polymers to achieve a nonadhesive, nonbiofouling surfaces such as PEG (polyethylene glycol), hydrogels (containing dextran), and PEO (polyethylene oxide). For example, ePTFE grafts have been coated with polypropylene sulphide (PPS)-PEG and evaluated in arteriovenous models. This study included heparinised and nonheparinised graft perfusion and evaluated cell adhesion and thrombus formation. No difference was observed in cell adhesion when compared with controls; however, the surface coating significantly decreased thrombus formation when used in conjunction with heparin. Dextrans (hydrophilic polysaccharides) show a similar effect to PEG with regard to protein adsorption. Dextrans, PEG, and PEO can be further chemically modified along the polymer backbone with cell-selective peptides to promote specific cell adhesion. Spin coating of the luminal surface of ePTFE was recently achieved using a biodegradable elastomer poly(1,8-octanediol citrate) (POC). The POC coatings had no effect on graft compliance and delayed thrombosis *in vitro* when compared with controls. This study highlighted that POC-ePTFE grafts maintained EC adhesion and proliferation of porcine cells similar to that of the native tissues, and within 10 days the EC was confluent, while only random patches were evident on ePTFE controls.

### 4.2. Biofunctionalisation

#### 4.2.1. Endothelialisation

 The endothelium is in intimate contact with the blood flow and consists of a single layer of EC, which functions as a dynamic organ and covers the entire surface of the circulating system from the heart to the smallest capillary. Endothelialisation of cardiovascular implants is considered most favourable as this would protect the vessels by producing natural biochemicals, for example, NO for vasoprotection. There is a great deal of research involved in inducing endothelialisation of cardiovascular implants and *insitu *endothelialisation is considered to be most favourable. This need for* insitu *endothelialisation has led to a considerable interest in stem cells which have the potential to induce endothelialisation. This interest in endothelial progenitor stem cells inturn has given rise to an exciting area of research on “stem cell technology” for vascular grafts.

Stem cells/EPC are on the threshold of realising their great potential in cardiovascular therapy and stem cell interactions with various biomaterials which have been extensively studied [30–34]. Cells are inherently sensitive to physical, biochemical, and chemical stimuli from their surroundings. Cells are in intimate contact with the ECM, which is formed from a complex connection of proteins, glycoproteins, and proteoglycans. The ECM provides not only structural support but also contains a reservoir of cell signalling motifs (ligands) and growth factors that guide cellular anchorage and behaviour. The local cell environment or “niche” provides defined environmental cues that determine cell-specific behaviour, including selective recruitment, proliferation, differentiation, and the production of the numerous proteins needed for hierarchical tissue organisation. The plethora of ECM compositions contain insoluble macromolecules fibrillar proteins (e.g., collagen) and glycoproteins (e.g., elastin, fibronectin, laminin) which interact with proteins on cell surfaces and soluble macromolecules such as growth factors. The organisation, density, spatial geometry, and biochemistry of these ECM components determine mechanical strength, cell response, and ultimately hierarchical tissue organization.

Features of the ECM such as nanoscale topography, optimised mechanical properties, and presentation of bioresponsive motifs have inspired multiple examples of biomaterials design for tissue engineering scaffolds. One strategy in vascular research is to present endothelium-derived macromolecules or their cell interacting domains onto vascular grafts to mimic these features of the ECM and to assist specific cell adhesion. Bioresponsive vascular grafts can target several biological processes to promote *insitu *endothelialisation including: (1) promoting the mobilisation of EPC from the bone marrow, (2) encouraging cell-specific (circulating EC, EPC, and/or stem cells) homing to the vascular graft site, (3) providing cell-specific adhesion motifs (peptides) on the vascular grafts (of a predetermined spatial concentration), and (4) directing the behaviour of the cells after adhesion to rapidly form a mature, fully functioning endothelium capable of self-repair.

Optimal cell attachment, migration, proliferation, and differentiation on a biomaterial require a surface which mimics the natural ECM. Natural ECM proteins range in diameter from 50–500 nm. The significance of mimicking the ECM, which facilitates the interactions with the cell receptors such as integrins, has been discussed. This also recognises the effect of peptides such as RGD [35], which are derived from functional domains of ECM components and their effects on enhancing accelerated endothelialisation. Nevertheless, nonreceptor-mediated interaction of ECM such as porosity, 3D spatial arrangement also has a great influence in cell interaction.

The microtopography of scaffold materials is not entirely ideal for vascular cells, particularly EC as they are naturally placed in a nanometre scale environment. Nanotopography surfaces created by surface roughening of a range of materials including polymers have shown enhanced cellular adhesion [36–40]. Recent reviews have discussed various nanotechniques which could potentially be applied to vascular graft engineering. Nanostructures has been shown to facilitate protein interactions which then promote cell adhesion [9, 41, 42]. Some of these proteins are selective such as vitronectin and fibronectin where they mediate enhanced vascular cell interactions with the polymer [37, 38]. Three main nanotechnology approaches, electrospinning [43, 44], self-assembly, and phase separation, help to create nanofibres and to create a nanoarchitecture bypass graft surface for optimal cell interactions [45].

In addition to mimicking an ECM, research has looked into antibody-mediated stem cell recruitment as a rather impressive approach in stem cell technology for applications in vascular graft endothelialisation. EC and EPC have been found to express CD34+, and therefore CD34+ antibodies can be attached onto bypass graft surfaces to facilitate interaction between graft and progenitor cells. Antihuman CD34 monoclonal antibodies (IgG2a, epitope class III) were immobilised to the ePTFE graft material (Orbus Medical Technologies) with a proprietary multistep process.

In additional, further studies have shown that superparamagnetic nanoparticles labelled endothelial cells can be used to obtain an endothelial cell lining, for instance, as on the luminal surface PTFE tubular grafts, coated with fibronectin with the aid of a customized electromagnet [46].

#### 4.2.2. Antithrombogenic Surfaces Independent of an Endothelium

 The cardiovascular protective role of the endothelium is recognized to be attributed to NO. Therefore, there is a great interest in inducing NO from cardiovascular implants and this has recently been reviewed in depth [47]. Recent reviews have also detailed numerous anticoagulant and antiplatelet agents that include, heparin, warfarin, hirudin, dipyridamole clopidogrel, aspirin, cilostazol, and glycoprotein IIb/IIIa inhibitors (abciximab, eptifibatide, tirofiban), which are clinically used in addition to their applications in engineered vascular graft surfaces [4, 48].

## 5. Summary and Future Perspective

Blood contacting materials, which are used to fabricate cardiovascular implants, are expected to preferably promote endothelial adhesion but resist other blood cell adhesion that can give rise to thrombosis and intimal hyperplasia. A greater understanding of the interactions of blood proteins with material surfaces will enable better designing of surfaces for blood contact. Surface modifications of materials will be highly influenced by nanotechnology as this enables to impart favourable properties without influencing the structural and mechanical properties of the base material, which forms the structure of a device of interest. It is also of significance that a particular surface modification should be always tailored to the implant of interest and should be tested for its efficacy in physiological haemodynamic conditions. We believe that a reasonable progress has been made in the search for optimal blood contacting materials, but research into NO eluting polymers, endothelialisation, and nanotechnology associated with surface modification of such materials may promise more sophisticated solutions in the quest for optimal blood compatibility.

## Figures and Tables

**Table 1:** Summary of the various modification techniques currently employed for optimising blood-material interactions [26].

Modification	Description
Physical immobilisation	Polymer gelling (growth factor mixed with the material in the liquid state and change temp, pH or ion concentration to obtain a gel with nanopores) Emulsion techniques (factors which are insoluble in aqueous solutions) High pressure gas foaming (incorporate GF into porous scaffolds, without the use of solvents)

Covalent modification	Surface distribution of ligands Distribution of ligands through the bulk of the material

Surface adsorption	Passive adsorption driven by secondary interactions between the molecule and the protein Self-assembled monolayers (SAMs) adsorption of the peptide (which is designed with hydrophobic tail and a spacer) from solution Microcontact printing of alkanethiol SAMs, photolithography (on hard materials), soft lithography (on elastomeric materials) Direct protein patterning: drop dispensing, microfluidic patterning

Crosslinking	Photo/chemical crosslinking

Altering surface wettability	Ion bombardment UV irradiation Exposure to plasma discharge

Altering surface roughness	Deposition of polymer films/islands, nanoparticles, metallographic paper or diamond paste polishing, sand blasting, photolithography, and e-beam etching
